# Practitioner perceptions of the feasibility of common frailty screening instruments within general practice settings: a mixed methods study

**DOI:** 10.1186/s12875-022-01778-9

**Published:** 2022-06-27

**Authors:** Rachel C. Ambagtsheer, Mavourneen G. Casey, Michael Lawless, Mandy M. Archibald, Solomon Yu, Alison Kitson, Justin J. Beilby

**Affiliations:** 1 National Health and Medical Research Council Centre of Research Excellence in Trans-Disciplinary Frailty Research to Achieve Healthy Ageing, GPO Box 2025, Adelaide, SA 5000 Australia; 2 Torrens University Australia, Adelaide, Australia; 3 Institute of Health and Wellbeing, Federation University Australia, Ballarat, Victoria Australia; 4 College of Nursing and Health Sciences, Flinders University, Adelaide, Australia; 5 College of Nursing, Rady Faculty of Health Sciences, University of Manitoba, Winnipeg, Manitoba Canada; 6 Adelaide Medical School, University of Adelaide, Adelaide, Australia

**Keywords:** Frailty, Aged, 80 and over, General practice, Primary care, Mass screening, Community health nursing

## Abstract

**Background:**

Frailty is a highly prevalent clinical syndrome increasing older people’s vulnerability to risk of adverse outcomes. Better frailty identification through expanded screening implementation has been advocated within general practice settings, both internationally and within Australia. However, little is known about practitioner perceptions of the feasibility of specific instruments, and the underlying motivations behind those perceptions. Consequently, the purpose of this study was to explore the attitudes and perceptions of a convenience and volunteer sample of Australian general practitioners (GPs) and practice nurses (PNs) towards common frailty screening instruments.

**Methods:**

The feasibility of several frailty screening instruments (PRISMA-7 [P7], Edmonton Frail Scale [EFS], FRAIL Questionnaire [FQ], Gait Speed Test [GST], Groningen Frailty Indicator [GFI], Kihon Checklist [KC] and Timed Up and Go [TUG]) to 43 Australian GPs and PNs was assessed. The study adopted a concurrent embedded mixed-methods design incorporating quantitative (ranking exercise) and qualitative (content analysis) data collection integrated during the analysis phase.

**Results:**

Practitioners assessed multi-dimensional instruments (EFS, GFI, KC) as having relatively higher clinical utility, better integration into existing assessment processes and stronger links to intervention over uni-dimensional (GST, TUG) and simple (FQ, P7) instruments.

**Conclusions:**

While existing frailty screening instruments show promise as an initial step in supporting better care for older people, all the included instruments were associated with perceived advantages and disadvantages. Ultimately, clinicians will need to weigh several factors in their selection of the optimal screening instrument. Further translational research, with a focus on contextual fit, is needed to support clinical decision-making on the selection of instruments for frailty screening.

**Supplementary Information:**

The online version contains supplementary material available at 10.1186/s12875-022-01778-9.

## Background

Frailty, a clinical syndrome in which older adults experience increased susceptibility to adverse outcomes due to exposure to external stressors [1–3], has received increasing research and clinical attention [4–6]. In 2013, an international consensus statement called for widespread frailty screening of all older people aged 70 years and over [7], later followed by international guidelines recommending opportunistic screening for all adults aged 65 years and over [8]. Within the UK, a position statement advocating frailty screening of older people at all routine health care encounters [9] and subsequent integration of systematic frailty identification and treatment was included within the 2017–2018 GP Contract [10]. In Australia, relevant guidelines, such as the Asia-Pacific Clinical Guidelines on the management of frailty has recommended routine screening of all older people aged 70 years and over [3], and the Royal College of General Practitioners’ (RACGP) Aged Care Clinical Guide, advocate assessing frailty annually [11]. However, little is known about practitioner attitudes towards specific screening instruments currently used within the primary care sector [12], with the exception of a few notable studies [13–20]. Where the feasibility of frailty screening instruments has been assessed, studies have tended to focus on accuracy, fit-for-purpose, dimensions/domains represented, time and equipment requirements [1, 19, 21, 22]. In particular, application of a mixed methods design to specifically explore general practitioner (GP) and practice nurse (PN) perceptions of frailty screening instruments and the reasons for those perceptions has been under-utilised. Consequently, the aim of this study was to explore the perceptions of Australian GPs and PNs towards the feasibility of selected frailty screening instruments currently in common use worldwide, using a mixed methods approach. Specifically, we sought to address this aim through the following research objectives:

1. Assess the relative perceived feasibility of instruments through implementation of a quantitative ranking process
2. Analyse potential differences in perceived feasibility by profession
3. Explore participant rationales for ranking order via qualitative methods

## Methods

### Study design

Data presented within this study were collected as part of a broader research project exploring stakeholder understandings and experiences of frailty and frailty screening [12]. A concurrent embedded mixed-methods research design was adopted [23]. A mixed-methods research design was thought particularly relevant to this study given that we sought both an objective ranking and a subjective explanation for the expressed ranked order. The research team adhered to the Good Reporting of A Mixed Methods Study (GRAMMS) principles in reporting the research [24]. The Torrens University Higher Research Ethics Committee (HREC 10/17) provided ethics approval. All participants gave written informed consent.

### Participants and setting

Study participants were GPs and PNs actively working in Australian general practice settings. Recruitment for the study proceeded in three phases. In the first phase, 22 South Australian GPs were purposively recruited for a balance across age, sex and rurality into a series of three focus groups conducted between September and December of 2016 (two face-to-face in Adelaide, one virtual using Zoom technology [25]) using a combination of snowball and convenience sampling. Further details on our recruitment strategy for GPs can be found in a previously published manuscript [26].

In the second phase, 16 PNs working in general practice were recruited for individual semi-structured interviews between March and August of 2017. Recruitment was conducted Australia-wide. An initial approach was made via South Australian Primary Health Networks (government-funded administration bodies) and was later expanded via an advertisement in an electronic newsletter targeted to primary health care providers. This sub-group will be referred to within this paper as ‘non-administering PNs.’

In the third phase, an additional five PNs who administered frailty screening instruments as part of a larger diagnostic test accuracy (DTA) study [27] were interviewed within two months of the DTA study completion by a member of the research team (RA). This sub-group will be referred to within this paper as ‘administering PNs.’ GP and non-administering PN participants received reimbursement for their time.

### Frailty screening instruments

As detailed further in a prior publication [28], the research team initially selected frailty screening instruments on the basis of a literature review, prioritising validity (sensitivity > = 0.6), appropriateness to context (in English, easily transferable to Australian setting) time to implement (≤20 min) and delivery method (administered rather than electronic). For the purpose of analysis, we grouped the instruments into three categories: *multi-dimensional* (instruments reflecting a multi-dimensional construct of frailty beyond just physical measures, structured into sub-domains), *simple* (instruments reflecting a multi-dimensional construct of frailty beyond just physical measures, but not structured into sub-domains) and *uni-dimensional* (instruments reflecting only a single, physical concept of frailty.)

#### Multi-dimensional

- Groningen Frailty Indicator (GFI), a 15-item multi-domain instrument with a frailty threshold of 4 or more points [29];
- Kihon Checklist (KC), a self-reported checklist consisting of 25 yes/no questions covering multiple domains, with 7 or more ‘yes’ questions indicating frailty [30, 31]
- Edmonton Frail Scale (EFS), a multi-domain instrument covering ten domains, scored from 0 to 17 with a frailty threshold set at 8 or more points [32].

#### Simple

- PRISMA-7, a 7-item questionnaire structured as yes/no responses, with three or more yes responses indicating frailty [33];
- FRAIL Scale Questionnaire (FQ), a 5-item questionnaire inclusive of questions on fatigue, resistance, ambulation, illness and loss of weight. Scaled from 0 to 5, with a threshold of 3 or more points indicating frailty [34].

#### Uni-dimensional

- Timed Up and Go, a timed rise from a seated position followed by a short walk over 3 m [35];
- Gait Speed (GST) measures the speed at which participants walk over a marked distance of 4 m (measured twice and averaged). A threshold of ≤0.8 m/s signifies frailty [36–38].

### Data collection

Prior to commencement of the focus groups and interviews, participants completed a short demographic survey either online for virtual sessions or on paper prior to attendance at in-person sessions. Participant locations were classified as ‘Major Cities’ or ‘Other’ on the basis of the Australian Bureau of Statistics Localities to Remoteness Area concordance (2016) [39]. All focus groups were facilitated by an experienced academic GP researcher (JB) and a member of the research team (RA). In addition, two researchers (SY, MA) attended individual focus groups as observers. Each focus group took between one and two hours (*M* = 101 minutes). Interviews with non-administering PNs were conducted virtually using Zoom technology by two researchers (MC, RA), with a mean length of 66 minutes. Interviews with administering PNs were conducted face to face by a member of the research team (RA) with a mean interview length of 32 minutes. Interview times for administering and non-administering PNs differed in length on average, as administering nurses were interviewed on site within the context of a busy GP clinic, allowing comparatively less time available for the interviews. The multi-disciplinary research team (inclusive of representation from the general practice, nursing and geriatrics disciplines) developed all interview and focus group guidelines collectively.

During the session, participants were given an overview of each instrument and asked to rank the frailty screening instruments. Specific statistics on the accuracy of each instrument were deliberately excluded from the information provided to encourage participants to consider feasibility independently of accuracy, although participants were informed prior to undertaking ranking that all instruments had been previously validated as screening instruments for frailty. Participants were then asked to rank the instruments in order of their perceived feasibility for application within the Australian primary care context, where 1 = “most feasible” and 7 = “least feasible”. Individual focus group members completed the ranking exercise independently and then discussed their preferences with the broader group. PNs discussed their preferences with the interviewer. GPs in FG1 (*n* = 6) did not discuss the KC during the focus group due to its later inclusion in the analysis. Two GPs submitted their ranking via email after the focus group concluded, leaving four with incomplete ranked data.

Focus groups were audio- and video-recorded using Zoom technology [40]. Interviews with non-administering PNs were audio- and video-recorded using Zoom, and for administering PNs, audio-recorded using an iPhone 7. All focus groups and interviews were transcribed by a professional third-party service.

### Data analysis

#### Quantitative

Categorical variables are reported as frequencies and percentages, with descriptive statistics generated to illustrate participant characteristics. Differences in instrument rankings by profession were assessed via Fisher’s exact test within a chi-square analysis with statistical significance set at *p* < 0.05. The analysis was performed using SPSS v25.

#### Qualitative

Transcripts were subjected to a qualitative, inductive content analysis [41] by two members of the research team (RA, MC) to elicit participant rationales for their chosen ranking order in the quantitative phase. Transcripts were read repeatedly by both researchers to gain familiarity with the data. In the first step, one focus group transcript was selected at random and independently coded by both researchers, then compared to generate an initial code list. The remaining transcripts were coded by a single researcher against the agreed code list, with the initial set of codes refined iteratively as they were applied. Once a final code list was discussed and agreed upon by the coding researchers, all transcripts were checked and re-coded against the agreed list of codes (RA). Results were initially shared with a third and fourth team member (ML, MA) and then with the broader research team to finalise development of themes and sub-theme. Themes and sub-themes were derived inductively, and were based in part on a consideration of whether participants mentioned an issue at least once in relation to an individual instrument (i.e. presence of a code), rather than frequency of mentions by individuals. An example drawn from the results illustrating the relationship of meaning units through categories, sub-themes and themes is shown in Table 1.

**Table 1:** Qualitative coding example showing relationship of theme, sub-theme, code and participant quotation

Theme	Subtheme	Code	Participant quotation
Feasibility for practice setting	Logistical aspects of clinical setting	Physical space as a barrier to implement-ation	“I don’t know that everybody is gonna have an accurate four-meter measurement area-that’s, you know, convenient and you know, not- a lot of nurses don’t work in a- they’re shoved in a corner somewhere and do not have a four-meter space in their consultation room. Not necessarily even in the hallway outside the consultation room. I just think, logistically it’s gonna be difficult.” (PN05)

#### Integration

Integration occurred at the interpretation phase of the analysis, where the qualitative content analysis was employed to give greater context to the results of the quantitative ranking exercise [23], and was primarily conducted by one researcher (RA) in consultation with the research team.

## Results

### Participant characteristics

Participant characteristics are shown in Table 2. A total of 43 clinical practitioners participated in the study including 21 PNs (48.8%) and 22 GPs (51.2%). Most were female (*n* = 34, 79.1%), worked in major cities (*n* = 31, 72.1%) and claimed over 10 years of clinical experience (*n* = 28, 65.1%).

**Table 2:** Participant characteristics (*n* = 43)

	GPs	PNs	Total
Characteristic	n (%)	n (%)	n (%)
Gender			
Male	9 (40.9)	0 (0.0)	9 (20.9)
Female	13 (59.1)	21 (100.0)	34 (79.1)
Age, years			
< 35	12 (54.5)	3 (14.3)	15 (34.9)
35 to 44	1 (4.5)	2 (9.5)	3 (7.0)
45 to 59	4 (18.2)	14 (66.7)	18 (41.9)
60+	5 (22.7)	2 (9.5)	7 (16.3)
Professional experience, years			
< 5	3 (13.6)	1 (4.8)	4 (9.3)
5 to 10	9 (40.9)	2 (9.5)	11 (25.6)
11 to 20	1 (4.5)	3 (14.3)	4 (9.3)
Over 20	9 (40.9)	15 (71.4)	24 (55.8)
Location			
Major cities	15 (68.2)	16 (76.2)	31 (72.1)
Other	7 (31.8)	5 (23.8)	12 (27.9)

### Quantitative results: feasibility rankings

Feasibility ranking scores for each test are summarised in Table 3. Table 4 shows the distribution of scores by instrument for selected placements. The table indicates that the order of ranking from most to least feasible as determined by top placing was 1) EFS, 2) KC, 3) GFI, 4)TUG, 5) P-7 and FQ, and 6) GST. Placement of instruments within the top three most feasible instruments followed a similar pattern. All seven instruments received a full range of ranking scores (1–7) except the GST, which was not nominated within a top two ranked position.

**Table 3:** Participant preferences for frailty screening instruments by instrument and preference order (*n* = 39)

Instrument	First preference		Second preference		Third preference		Fourth preference		Fifth preference		Sixth preference		Seventh preference	
	n	%	n	%	n	%	n	%	n	%	n	%	n	%
EFS	15	(38.5)	12	(30.8)	3	(7.7)	5	(12.8)	1	(2.6)	2	(5.1)	1	(2.6)
KC	10	(25.6)	7	(17.9)	9	(23.1)	5	(12.8)	3	(7.7)	4	(10.3)	1	(2.6)
GFI	4	(10.3)	9	(23.1)	10	(25.6)	5	(12.8)	8	(20.5)	1	(2.6)	2	(5.1)
TUG	4	(10.3)	0	(0.0)	7	(17.9)	6	(15.4)	6	(15.4)	13	(33.3)	3	(7.7)
P7	3	(7.7)	3	(7.7)	4	(10.3)	4	(10.3)	7	(17.9)	5	(12.8)	12	(30.8)
FQ	3	(7.7)	8	(20.5)	5	(12.8)	9	(23.1)	7	(17.9)	4	(10.3)	4	(10.3)
GST	0	(0.0)	0	(0.0)	1	(2.6)	5	(12.8)	7	(17.9)	10	(25.6)	16	(41.0)
TOTAL	39	(100.0)	39	(100.0)	39	(100.0)	39	(100.0)	39	(100.0)	39	(100.0)	39	(100.0)

*Abbreviations: P7* PRISMA-7, *FQ* Frail Questionnaire, *GST* Gait Speed Test, *GFI* Groningen Frailty Indicator, *EFS* Edmonton Frail Scale, *KC* Kihon Checklist, *TUG* Timed Up and Go

NB: Tables 3 and 4 exclude 4 GPs missing the KC from their rankings

**Table 4:** Participant rankings for frailty screening instruments by instrument and ranked position (n = 39)

Instrument	First place ranking (most feasible)	Top third ranking	Bottom third ranking	Last place ranking (e.g. least feasible)
	%	%	%	%
EFS	34.9	69.8	9.3	2.3
KC	23.3	60.5	18.6	2.3
GFI	9.3	53.5	25.6	4.7
TUG	9.3	25.6	51.2	7
P7	7	23.3	55.8	27.9
FQ	7	37.2	34.9	9.3
GST	0	2.3	76.7	37.2

*Abbreviations: P7* PRISMA-7, *FQ* Frail Questionnaire, *GST* Gait Speed Test, *GFI* Groningen Frailty Indicator, *EFS* Edmonton Frail Scale, *KC* Kihon Checklist, *TUG* Timed Up and Go

NB: Tables 3 and 4 exclude 4 GPs missing the KC from their rankings

No significant difference between professions was observed with respect to propensity to rank a comprehensive (EFS/KC/GFI), simple (FQ/P7) or unidimensional (GST/P7) instrument in first place, top three, bottom three or last place, as determined by Fisher’s exact test. Administering nurses (*n* = 5) were significantly more likely (*p* = 0.047) than other participants to nominate simple instruments (P7/FQ) as one of their top three rankings, but no other significant differences were found. Lastly, non-metropolitan participants were significantly more likely to nominate a unidimensional instrument within their top three rankings than were metropolitan participants (*p* = 0.017), but not as their first ranking overall.

### Qualitative content analysis: perceived properties of screening instruments

The content analysis of focus groups and interview transcripts generated 81 unique codes, 9 sub-themes and 3 themes. Key themes identified were ‘Support for clinical decision making’, ‘Feasibility for practice setting’ and ‘Support for patient-centred care’ (Table 5).

**Table 5:** Qualitative themes and sub-themes

Theme	Sub-Theme
Support for clinical decision making	Practitioner confidence in instrument results
	Offers new insights into the patient condition
	Links to intervention
Feasibility for practice setting	Ease of administration
	Logistical aspects of clinical setting
	Alignment with practice routines
Support for patient-centred care	Acceptability to patients
	Preservation of duty of care (dignity and safety)
	Support for effective communication

#### Theme 1: support for clinical decision making

Participants routinely accounted for their evaluations of instruments with reference to the perceived validity (e.g. how well they measured what they purported to measure), often mentioning face validity rather than other aspects (e.g. predictive validity). As one participant noted of the GFI: *“It’s almost like this mini holistic look, which I think with frailty … my perception has been, that it is something holistic”* (PN04). The inclusion of mental health and psychosocial elements was seen as critical by many (44.2%), but physical mobility (18.6%), functional ability, medications and cognition (all at 16.3%) were also valued. Likewise, where participants perceived a relevant assessment component as missing (58.1%), concerns were raised that the instrument was invalid. This was most often specified in relation to unidimensional instruments such as the GST (20.9%) and TUG (37.2%), which participants felt focused only on physical frailty, along with the FQ (23.3%). As one GP commented in relation to TUG, *“… (you’re) missing this social sort of frailty and the cognitive frailty and potentially other frailties*” (FG02P5).

Participants also raised concerns about bias, overwhelmingly in regard to a negative perception of self-reported items and the implication that patients would provide inaccurate responses. About one-third of participants felt the use of self-reported items would lead to patients providing dishonest responses (32.6%). As one PN stated, *“patients will lie, they’re very good at it, because they are worried about what you may or may not do…”* (PN04). Participants also worried that self-reported instruments were open to misinterpretation by patients, for example, not understanding terminology used (30.2%), or that patients may not know the answer to the question (30.2%). Other related issues identified included illiteracy and low English proficiency. Beyond self-reporting, 11.6% of participants expressed concerns that instruments would not be consistent over time (i.e., poor test-retest reliability).

Most (67.4%) participants viewed screening instruments favourably if they perceived them to offer new insights into the patient’s condition, especially in relation to multi-domain instruments. As one nurse remarked of the EFS, *‘It’s obviously a lot more complex, and it’s picking up a lot of different areas. So, you’re getting a lot more information from your patient’* (PN14). In contrast, views were mixed on unidimensional instruments. For some (27.9%), the prospect of an objective frailty score offered advantages, such as tracking patient progress over time, or comparing results between administrators. However, others (55.8%) felt that formal administration of a screening instrument would not add any value in the case of patients who they already knew or suspected to be frail, as the screening would duplicate activities already performed on either an informal (or much less often, formal) basis, e.g. walking across a room. This view was more frequently expressed for physically-oriented instruments such as the GST (23.3%) and TUG (20.9%) in contrast to others (4.7–11.6%). As one administering nurse remarked: *“…I don’t necessarily think you need to time people, I think you can see how frail they are unless you really don’t know them.”* (PN20).

Lastly, for just over a quarter of participants (27.9%), a clear link to intervention based on the screening instrument was an advantage, in the sense of giving a practical indication of next steps. Multi-domain instruments such as the EFS (14.0%) and GFI (9.3%) were most frequently identified as useful in this manner. As one PN mentioned, *“…when you’ve got multiple areas, you can possibly respond with one action that might cover, you know, a few areas…it will really outline where they are frail and how to respond.”* (PN04). However, some participants felt that there might be a role for less comprehensive instruments in a two-step process of initial screening and later assessment. One GP remarked, *“… a quick screening tool would be the best option; so if you have something that would … get your hackles raised and the alarm bells going, to prompt you to give a more comprehensive one, that would probably work quite well”* (FG03P2). In these cases, practice software was sometimes proposed to notify the GP that a patient needed following up.

#### Theme 2: feasibility for practice setting

Most participants (83.7%) identified ease of administration as a perceived advantage, as typified by the expression “*short and sweet*” used by one nurse in relation to the P7 (PN07). Over half of all participants characterised instruments as being easy (53.5%), quick (48.8%) or simple (30.2%). Key disadvantages identified were excessive time to complete (60.5%), high degree of complexity (25.6%) or excessive length (18.6%). Views were mixed regarding specific instruments; while the P7 and FQ were viewed almost exclusively as easy (41.9 and 37.2% respectively) rather than difficult (2.3 and 7.0%), almost equal proportions of participants viewed the EFS, GFI, GST, and KC as either easy or difficult to administer.

Logistics were also considered by participants, where disadvantages of instruments were identified almost twice as frequently (51.2%) as advantages (23.3%). In our analysis, logistics referred to organisational or operational aspects of implementing screening, such as space, time, equipment, and personnel. Logistics were most viewed as disadvantageous for instruments incorporating timed physical exercises (GST: 34.9%, TUG 25.6%, EFS 18.6% of participants), particularly relating to space but also to equipment. As one nurse noted: *“I can’t even imagine where we would find a well-lit unobstructed and clearly indicated (space) … that’s four meters in length within our practice”* (PN15). However, some nurses (*n* = 5) felt space constraints could be met by moving patients into the corridor or waiting room. Lastly, the KC was perceived as problematic for rural/remote clinics by six participants due to its inclusion of public transport use, which is not readily available outside Australian urban areas.

Lastly, alignment with existing practice routines was identified by just over three-quarters (76.7%) of participants as an advantage, especially in relation to fit with existing assessments. Instruments perceived as aligning well with existing assessments were the KC (27.9%), the EFS (23.3%) and the GFI (20.9). In particular, participants noted how the content of the multi-dimensional instruments mirrored similar questions asked during the assessments. One nurse remarked in regard to the EFS, *“I think it would be something that we’d easily integrate in to what we already do with our over 75 assessments because … the questions sort of cross over anyway”* (PN16). Instruments perceived as aligning less well were GST and TUG (both 11.6%), both of which were perceived to be outside the scope of general practice.

Two time-saving options proposed by participants included having nurses do most of the screening (30.2%) or patients self-administer screening instruments in the waiting room (32.6%), thus minimising GP involvement. Nurse-led screening and assessment was viewed by both GPs and PNs as an extension of work flows already present within the practice. For example, one GP stated in relation to the EFS, *“I feel like this is one that I’d get my practice nurse to do because he can then have a quick look and see in which points they scored well and didn’t score well.”* (FG02P2). Often, a two-step process was proposed in which nurses performed an initial screen and then briefed the doctor prior to a more comprehensive assessment. Self-administration was most frequently raised as an advantage regarding the KC (16.3%) but was also raised for the EFS, P7, GFI and FQ.

#### Theme 3: support for patient-centred care

Participants also spoke about feasibility in terms of how well they envisioned patients would react to individual instruments. Views were mixed on how acceptable instruments would be to patients. Only one instrument (P7) received substantially more identification as highly acceptable rather than low. Factors identified as potential disadvantages were unnecessary repetition, awkward phrasing, excessive length or seemingly self-evident tasks, e.g. EFS clock test. As one administering PN noted, *“Some of them got really offended with this clock… ‘…What do you think we are, kids? We know how to tell the time!’”* (PN20). Almost a quarter of participants (23.3%) raised the prospect of patients experiencing screening as physically challenging (GST, TUG, EFS), especially if impacted by diseases such as Rheumatoid Arthritis, or who were very frail. Approximately one-fifth (20.9%) labelled instruments as mentally challenging (EFS, GFI, KC), especially where patients were cognitively impaired or experiencing memory difficulties.

A second sub-theme related to the perceived ability of instruments to support effective communication between patients and healthcare providers. A key disadvantage related to the inclusion of closed-ended questions (32.6%). As one nurse stated of the KC, *“I would assume that hopefully the person doing this doesn’t just tick yes or no and that they’d ask a bit more information. So, if somebody said, “Yes, I’ve fallen over in the past year“, I would hope that they asked, you know, how? Where? Why?”* (PN08). However, three GPs viewed close-ended questions as advantageous, especially in time-limited situations. Approximately one-third of participants viewed instruments positively if they supported communication with patients (32.6%). For example, one nurse said of the EFS, “*It gives patients or clients the chance to comment and to give you feedback… I would happily use that as a basis for guiding a conversation*” (PN15). Four participants felt that objective frailty measurement scores based on timed physical exercises (GST, TUG) might be helpful motivation for patients to enact healthy behaviours.

Endangering duty of care was raised as a disadvantage by approximately one-quarter (23.3%) of participants, in the sense of identifying instruments that may threaten patients’ dignity or safety, commonly in relation to GST (20.9%) and TUG (11.6%). Because such instruments require a minimum space to administer (3–4 m), participants highlighted the implication that space constraints would mean administration in the waiting room in front of other patients. This was viewed as unacceptable due to the perceived falls risk and the need to preserve the privacy of patients. As one nurse remarked, *“I can’t see it being used in general practice … because of finding a place they can walk four meters … we’ve got areas, but they’re waiting room areas, and I’d feel a bit horrible making a patient …totter down our waiting room being watched by our patients...”* (PN03) However, two participants felt waiting room administration would be acceptable. A small number of participants highlighted potential negative impacts of screening on patient’s mental health, due to the nature of items included within several of the multi-dimensional instruments, especially the GFI.

## Discussion

Our study aimed to explore the perceptions of Australian GPs and PNs towards the feasibility of frailty screening instruments, with specific attention to factors influencing perceptions. Our inductive analysis generated three themes and several associated sub-themes. We found that three key factors drove clinician perceptions of screening instruments: 1) the ability to support more effective clinical decision-making through improved confidence in screening results, contributing useful new information about patients or guiding intervention efforts; 2) fit for setting, including ease of administration, good logistical fit and alignment with practice routines; 3) patient-centredness, including being acceptable to patients, supporting effective communication and preserving patients’ safety and privacy.

It is often asserted within the frailty literature that GPs need access to rapid, simple instruments for screening [42–44]. However, participants consistently ranked comprehensive, multi-dimensional instruments such as EFS, GFI and KC over simpler instruments (FQ, P7) and unidimensional instruments (GST, TUG), both of which were perceived as less clinically useful, and in the case of GST and TUG, more burdensome for patients. The distinction between rankings for multi-dimensional vs. uni-dimensional instruments in particular is reflective of a broader conceptual disagreement within the frailty literature that is yet unfolding [45] – namely, the question of whether frailty is primarily a physical (the Frailty Phenotype model [46]) or multi-dimensional (the Deficit Accumulation, or Frailty Index, model [47]) phenomenon. Indeed, an aligned qualitative analysis indicated that GPs primarily viewed frailty as a multi-dimensional concept [26]. Administering PNs were the only sub-group of participants to rank simple instruments higher than other types, presumably because they represent a trade-off between comprehensiveness and speed of administration, but given the small number of participants in this group, this result should be interpreted with caution. It may be that the Australian context, in which comprehensive, annual health assessments are provided to all older people aged 75 years and older [48], allows for practitioners to allocate relatively more time to screening than other contexts where such opportunities are not as readily funded. The distinction between screening and assessment may also be of relevance here [22]; some of the rapid instruments perceived by participants as not offering sufficient links to intervention, such as the P7 and FQ, are perhaps more useful as the initial step in a two-step process of screening and subsequent assessment. Although some participants acknowledged this, there was also a sense that two-step screening and assessment was potentially doubling-up where one-step might do.

It should be noted that support for multi-dimensional instruments was not unilateral and that concerns regarding acceptability to patients were identified for even the most highly rated instruments, e.g. the administration of sensitive psycho-social items within the GFI, a finding supported by prior research [49], and the difficulty of administering the ‘clock test’ within the EFS. It may be that participatory methods, such as co-design of instruments with participant groups including both clinicians and patients, offer a promising means of addressing these issues in the future.

### Strengths and limitations

Ours is among the first studies, to our knowledge, to comprehensively explore GP and PN perceptions of the feasibility of a range of common frailty screening instruments using a mixed methods design. A strength of our study is the inclusion of participants across age ranges and rurality, a factor that enabled us to highlight potential implementation issues with some instruments in non-urban contexts, such as the inclusion of questions about frequency of public transport use in areas where public transport is limited. Further, the adoption of a mixed methods design allowed us to go beyond simple ranked perceptions of the feasibility of the instruments to unpack the considerations that participants weighed when making their judgements.

However, there were some limitations. Our study focused on provider perceptions, which necessarily limited the scope of the feasibility components analysed [50], and our study analysed feasibility independently of accuracy. Few participants had direct prior experience of the instruments, with the exception of the five administering PNs. More studies are needed to explore how implementation of frailty screening instruments will work in clinical practice, specifically the experience of professional groups other than PNs. The recruitment methods employed (snowball and convenience sampling), although useful in recruiting professionals from often hard-to-reach groups, had potential for increasing selection bias. For this reason, we do not claim generalisability of our results to the Australian clinical workforce. For practical reasons, our data collection method included both focus groups (GPs) and interviews (PNs), resulting in somewhat richer transcripts for PNs compared with GPs. Social desirability bias may have influenced some participants’ perspectives within the focus groups, where senior academic GPs were often present (either as facilitator or participants) [26]. Lastly, the GPs in FG1 did not discuss the KC during their focus group; instead, two offered their perspectives via email after the fact. This may have had the impact of reducing the total volume of discussion included about the KC slightly.

## Conclusion

Frailty screening within general practice contexts, followed by timely intervention, shows potential for driving more person-centred and responsive care for older people [8, 19]. However, to be implemented effectively, screening instruments must be both accurate and feasible within primary care settings. This study has explored clinician viewpoints on the feasibility of instruments currently within wide use, highlighting preferences for multi-dimensional instruments with relatively high perceived clinical utility, feasible fit with the clinical setting and minimal negative impact on patients. However, clinician concerns regarding implementation of frailty screening were also noted, with some noting the potential to expose patients to undue stress or burden. In future, practitioners will need to balance considerations about the accuracy of the instruments against fit with context and perceived patient benefit in their selection of instruments.

## Supplementary Information

**Additional file 1.**

## Data Availability

The datasets generated and/or analysed during the current study are not publicly available for reasons of confidentiality but are available from the corresponding author on reasonable request.
