# Supplementary material for: Practitioner perceptions of the feasibility of common frailty screening instruments within general practice settings: a mixed methods study
Source: BMC Prim Care. 2022 Jun 27;23:160. doi: 10.1186/s12875-022-01778-9 (PMC9235102; doi:10.1186/s12875-022-01778-9)
Supplement: Supplementary file 1 — Additional file 1. [file 12875_2022_1778_MOESM1_ESM.docx]

Supplement 1. Themes and sub-themes in the data by instrument: number (%) of participants identifying each sub-theme

|  | Instrument: number (%) of participants identifying sub-theme) | | | | | | | | | | | | | | | | | | | | | |
| --- | --- | --- | --- | --- | --- | --- | --- | --- | --- | --- | --- | --- | --- | --- | --- | --- | --- | --- | --- | --- | --- | --- |
| Theme/Sub-Theme | EFS | | FQ | | | GFI | | | GST | | | KC | | | P7 | | | TUG | | | TOTAL (n=43) | |
| Theme: Support for clinical decision making | | | | | | | | | | | | | | | | | | | | | | |
| Practitioner confidence in the instrument (-) | 13 | (30.2) | 28 | (65.1) | 19 | | (44.2) | 13 | | (30.2) | 12 | | (27.9) | 28 | | (65.1) | 20 | | (46.5) | 41 | | (95.3) |
| Practitioner confidence in the instrument (+) | 23 | (53.5) | 9 | (20.9) | 19 | | (44.2) | 2 | | (4.7) | 14 | | (32.6) | 8 | | (18.6) | 6 | | (14.0) | 34 | | (79.1) |
| Does not support new insights into patient condition (-) | 3 | (7.0) | 4 | (9.3) | 2 | | (4.7) | 13 | | (30.2) | 6 | | (14.0) | 5 | | (11.6) | 10 | | (23.3) | 28 | | (65.1) |
| Supports new insights into patient condition (+) | 10 | (23.3) | 11 | (25.6) | 5 | | (11.6) | 9 | | (20.9) | 10 | | (23.3) | 4 | | (9.3) | 14 | | (32.6) | 29 | | (67.4) |
| Poor links to intervention (-) | 2 | (4.7) | 2 | (4.7) | 1 | | (2.3) | 2 | | (4.7) | 2 | | (4.7) | 1 | | (2.3) | 0 | | (0.0) | 8 | | (18.6) |
| Good links with intervention (+) | 6 | (14.0) | 3 | (7.0) | 4 | | (9.3) | 0 | | (0.0) | 3 | | (7.0) | 1 | | (2.3) | 1 | | (2.3) | 12 | | (27.9) |
| Theme: Feasibility for practice setting | | | | | | | | | | | | | | | | | | | | | | |
| Low ease of administration (-) | 18 | (41.9) | 3 | (7.0) | 13 | | (30.2) | 17 | | (39.5) | 9 | | (20.9) | 1 | | (2.3) | 6 | | (14.0) | 32 | | (74.4) |
| High ease of administration (+) | 15 | (34.9) | 16 | (37.2) | 12 | | (27.9) | 17 | | (39.5) | 9 | | (20.9) | 18 | | (41.9) | 13 | | (30.2) | 36 | | (83.7) |
| Logistical aspects of clinical setting (-) | 8 | (18.6) | 1 | (2.3) | 1 | | (2.3) | 15 | | (34.9) | 5 | | (11.6) | 1 | | (2.3) | 11 | | (25.6) | 22 | | (51.2) |
| Logistical aspects of clinical setting (+) | 2 | (4.7) | 3 | (7.0) | 4 | | (9.3) | 3 | | (7.0) | 1 | | (2.3) | 2 | | (4.7) | 1 | | (2.3) | 10 | | (23.3) |
| Poor alignment with practice routines (-) | 1 | (2.3) | 1 | (2.3) | 3 | | (7.0) | 4 | | (9.3) | 0 | | (0.0) | 1 | | (2.3) | 4 | | (9.3) | 11 | | (25.6) |
| Good alignment with practice routines (+) | 20 | (46.5) | 10 | (23.3) | 13 | | (30.2) | 7 | | (16.3) | 14 | | (32.6) | 8 | | (18.6) | 8 | | (18.6) | 33 | | (76.7) |
|  | | | | | | | | | | | | | | | | | | | | | | |
|  | Instrument: number (%) of participants identifying sub-theme) | | | | | | | | | | | | | | | | | | | | | |
| Theme/Sub-Theme | EFS | | FQ | | | GFI | | | GST | | | KC | | | P7 | | | TUG | | | TOTAL | |
| Theme: Support for patient-centred care | | | | | | | | | | | | | | | | | | | | | | |
| Low acceptability to patients (-) | 9 | (20.9) | 6 | (14.0) | 11 | | (25.6) | 12 | | (27.9) | 5 | | (11.6) | 1 | | (2.3) | 10 | | (23.3) | 26 | | (60.5) |
| High acceptability to patients (+) | 8 | (18.6) | 3 | (7.0) | 4 | | (9.3) | 1 | | (2.3) | 4 | | (9.3) | 7 | | (16.3) | 2 | | (4.7) | 17 | | (39.5) |
| Does not preserve duty of care (-) | 0 | (0.0) | 0 | (0.0) | 1 | | (2.3) | 9 | | (20.9) | 1 | | (2.3) | 0 | | (0.0) | 5 | | (11.6) | 10 | | (23.3) |
| Preserves duty of care (+) | 0 | (0.0) | 0 | (0.0) | 0 | | (0.0) | 0 | | (0.0) | 0 | | (0.0) | 0 | | (0.0) | 0 | | (0.0) | 0.0 | | (0.0) |
| Does not support effective communication (-) | 6 | (14.0) | 6 | (14.0) | 11 | | (25.6) | 1 | | (2.3) | 8 | | (18.6) | 5 | | (11.6) | 2 | | (4.7) | 20 | | (46.5) |
| Supports effective communication (+) | 5 | (11.6) | 1 | (2.3) | 10 | | (23.3) | 1 | | (2.3) | 12 | | (27.9) | 2 | | (4.7) | 2 | | (4.7) | 22 | | (51.2) |

## Supplement 2: Question Guide: GP Focus Groups and PN Interviews

Part 1: Perceptions of, and Attitudes to, Frailty and Frailty Screening

What is your understanding of the concept of frailty? How would you define it from a clinical perspective?

When would you say that someone is frail?

What is your understanding of how frailty develops? How does it progress?

What, if anything, can be done to prevent people from becoming frail? What can be done once a person is frail?

What can GPs (and other HCPs) do to improve care of pre frail and frail persons?

Can you describe your attitude towards the concept of frailty screening? Under what circumstances if any do you feel it would be useful?

What experience if any with frailty screening have you had in your practice?

If a tool indicated a patient to be frail, what would the logical next steps be for you?

Part 2: Views on Selected Frailty Screening Tools

<Researcher presents information on each tool in turn. After each tool is presented, the following questions are asked for either group or individual discussion>

Focusing on Tool X now:

1. What do you think are some of the advantages if any of using Tool X for frailty screening within the general practice setting?

2. What do you think are some of the disadvantages if any of using Tool X for frailty screening within the general practice setting?

## Supplement 3: Frailty Screening Instrument Ranking Sheet

Instruction:

Please rank the tools listed below from 1 to 7 in order of their perceived feasibility for implementation within the Australian general practice setting, where 1 is *most feasible* and 7 is *least feasible*.

Please use all numbers from 1 to 7 and do not repeat any numbers twice. The tools have been presented in a random order.

| **Tool** | **Rank** |
| --- | --- |
| Kihon Checklist |  |
| Gait Speed Test |  |
| FRAIL Questionnaire |  |
| The Edmonton Frail Scale |  |
| Groningen Frailty Index |  |
| PRISMA-7 |  |
| Timed Up and Go |  |

Please comment on your rationale for the ranking order.

_______________________________________________________________________________________________________________________________________________________________________________________________________________

## Supplement 4: Results, Instrument Ranking by Profession

|  | GP | PN | p |
| --- | --- | --- | --- |
|  | n (% of GPs) | n (% of PNs) |  |
|  |  |  |  |
| Multi-Dimensional as First Ranked | 17 (77.3%) | 16 (76.2%) | 1.000 |
| Multi-Dimensional as Top 3 Ranked | 21 (95.5%) | 19 (90.5%) | 0.607 |
| Multi-Dimensional as Last Ranked | 2 (9.1%) | 2 (9.5%) | 1.000 |
|  |  |  |  |
| Uni-Dimensional as First Ranked | 3 (13.6%) | 1 (4.8%) | 0.607 |
| Uni-Dimensional as Top 3 Ranked | 7 (31.8%) | 4 (19.0%) | 0.488 |
| Uni-Dimensional as Last Ranked | 11 (50%) | 10 (47.6%) | 1.000 |
|  |  |  |  |
| Simple as First Ranked | 2 (9.1%) | 4 (19.0%) | 0.412 |
| Simple as Top 3 Ranked | 11 (50%) | 13 (61.9%) | 0.543 |
| Simple as Last Ranked | 9 (40.9%) | 9 (42.9%) | 1.000 |
